# Relationships Between Vertical Jump and Full Squat Power Outputs With Sprint Times in U21 Soccer Players

**DOI:** 10.2478/v10078-011-0081-2

**Published:** 2011-12-25

**Authors:** Manuel López-Segovia, Mário C. Marques, Roland van den Tillaar, Juan J González-Badillo

**Affiliations:** 1 Research Section, Murcia Soccer Federation, Murcia, Spain; 2 Department of Exercise Science, University of Pablo de Olavide, Seville, Spain; 3 Department of Sport Sciences, University of Beira Interior, Covilhã, Portugal; 4 Research Centre for Sport, Health and Human Development, Vila Real, Portugal; 5 Department of Teacher Education and Sports of Sogn and Fjordane University College, Norway

**Keywords:** strength, physical capacity, vertical jump, full squat, team sports

## Abstract

The aim of this study was to assess the relationship between power variables in the vertical jump and full squat with the sprint performance in soccer players. Fourteen under-21 soccer players were evaluated in two testing sessions separated by 7 days. In the first testing session, vertical jump height in countermovement was assessed, and power output for both loaded countermovement jump (CMJ_L_) and full squat (FS) exercises in two progressive load tests. The second testing session included sprinting at 10, 20, and 30m (T_10_, T_20_, T_30_, T_10–20_, T_10–30_, T_20–30_). Power variables obtained in the loaded vertical jump with 20kg and full squat exercise with 70kg showed significant relationships with all split times (r=−0.56/–0.79; p≤ 0.01/0.01). The results suggest that power produced either with vertical jump or full squat exercises is an important factor to explain short sprint performance in soccer players. These findings might suggest that certain levels of neuromuscular activation are more related with sprint performance reflecting the greater suitability of loads against others for the improvement of short sprint ability in under-21 soccer players.

## Introduction

Soccer is the world’s most popular sports game. Because of its popularity, many studies have been conducted in an attempt to understand the fundamental skills required by the soccer player (Wisloff, 1998; Cometti, 2001; Wisloff, 2004; Chelly, 2009; Meylan and Malatesta, 2009; Mújica, 2009; Requena, 2009). Unfortunately, the scientific understanding of this issue remains to lag behind its practice, with most participants acquiring skills through individual experience rather than research-based instruction.

Many sports depend heavily upon muscular strength and power especially at competition level (Baker, 2001; Haff, 2001), soccer being no exception (Wisloff, 2004). During a soccer match, lower body power is important for executing different activities as stopping and changing running speed as well as direction (Wisloff, 1998; Hoff and Helgerud, 2004; Thorlund, 2009). Although power performance has been studied in soccer (Wisloff, 1998; Wisloff, 2004), the relationships between lower body muscular power and linear sprint in soccer players have not been of primary researchers’ interest. Additionally, mostly isokinetic or isoinertial dynamometry has been used to elucidate the associations between strength/power measures and sprinting performance (Sleivert and Taingahue, 2004). Several studies have examined the possible associations between sprint ability and distinct strength and power measures in isoinertial exercises (Young, 1995; Wisloff, 1998; Baker and Nance, 1999; Henessy and Kilty, 2001; Sleivert and Taingahue, 2004; Cronin and Hansen, 2005; Marques and González-Badillo, 2006; Harris, 2008a; Requena, 2009; Habibi, 2010). In terms of lower body exercises, squat and squat vertical jump movements have been systematically used in order to explain sprint performance (Wisloff, 1998; McBride, 2002; Sleivert and Taingahue, 2004; Wisloff, 2004; Cronin and Hansen, 2005, Harris, 2008a; Harris, 2008b; González-Badillo and Marques, 2010), but determining the exact associations between these exercises and short sprinting ability has proved elusive. Indeed, for many sports activities, such as tennis, squash, and basketball, the athletes never attain maximum velocity during sprinting. Thus, the velocity over the first steps (first-step quickness) and the ability to rapidly increase velocity (acceleration) would be considered of greater importance to successful performance (Sleivert and Taingahue, 2004; Dellal, 2011). During a soccer match, sprints most frequently occur over very short distances while standing, stopping and changing the direction of movement (Di Salvo, 2009; Bradley, 2009; 2010). Thus, the acceleration phase is of major importance to soccer players. Moreover, little attention has been given to develop or refine new methods to improve soccer specific acceleration. It is commonly accepted that squatting and jumping are two important exercises to improve lower body strength, power and speed (Wisloff, 1998; Sleivert and Taingahue, 2004; Harris, 2008a; Requena, 2009). Previous investigations have shown significant associations between these two exercises and short sprint velocity (Sleivert and Taingahue, 2004; Harris, 2008a). However, the results of literature relating isoinertial assessment and sprint performance in team sports have been contradictory. Cronin and Hansen (2005) failed to find any relationship between sprint time and measurements of maximal strength, but reported weak correlations (r=−0.43 to −0.66) between vertical jump performance with 5, 10, and 30-m sprint times in professional rugby players (23.2 ± 3.3 years). In contrast, others showed moderate to strong relationships between squat performance and 10-m sprint times (r= 0.94), 30-m sprint times (r= 0.71), and jumping height (r= 0.78) (Wisloff, 1998). These results illustrate the difficulty in identifying how performances in various field tests can be related to one another.

Exercise intensity is acknowledged as an important variable related to changes in strength (ACSM Position stand, 2009) and has been commonly identified with relative load (% of one maximum repetition: 1RM) or with the maximum number of repetitions that can be performed with a given submaximal weight (XRM). However, to our best knowledge, no study prior to ours has examined the relationships between strength parameters with submaximal and absolute loads, without the evaluation of 1RM or XRM, and short sprint performance in soccer players. The understanding of these relationships may offer greater insight into the underlying determinants of short sprint performance and offers valuable information to improve the training process in soccer players.

Therefore, the purpose of this study was to assess the relationship between power variables in the vertical jump, full squat and sprinting performance in U-21 soccer players. It was hypothesized that the power for both exercises would be significantly related to sprint acceleration, but loaded vertical jump power would show a stronger relationship to sprint performance than traditional full squat power because it was a more sprint specific exercise.

## Material and Methods

### Study design

The present study used a cross-sectional experimental design to examine the relationship between muscle power and sprinting variables in a group of amateur (U-21) soccer players. Subjects were acquainted with all test procedures four weeks before the measurements were applied. All testing was completed at the end of a resistance-training period, in the middle of the competitive phase. This entailed a resistance-training program twice per week. The program included the full squat, the countermovement jump, and sprinting exercises. All the soccer players were well conditioned and familiar with all the testing exercises, which they had been performing regularly as part of training sessions.

### Subjects

Fourteen U21 soccer players (age 20.14 ± 0.4yr, body mass 75.5 ± 7.7 kg, body height 1.79 ± 0.10m) participated in the study. All participants belonged to the same sports club. Before commencing the experiment all subjects underwent a physical examination and were cleared of any medical disorders that might limit full participation in the study. The study was approved by the Research Ethics Committee. The subjects received information about the characteristics and goals of the study, the procedures, free participations, possibility of quitting at any moment, and confidentiality of data. Written informed consent was obtained from the subjects before beginning the study.

### Procedures

Participants were familiar with the testing procedures since they performed the exercises as part of their normal training routine (one or two weekly sessions). Following a standardized warm-up the subjects performed countermovement jumps, full squat, and sprinting exercises. Measurements were assessed in two sessions separated by 7 days amongst all participants. The first testing session included only countermovement jumps and full squats, whereas the second session accessed the sprint performance. During the execution of these tests, the players were verbally encouraged to give their maximal effort.

### Testing

Power was measured through the following tests: countermovement jump (CMJ), and both CMJ and full squat with external load on the Smith machine (Fitness Line Peroga, Murcia, Spain). The vertical jump height was measured using a trigonometric carpet (Ergo Jump Bosco System, S. Rufina di Cittaducale, RI, Italy). Subjects began from a standing position, performed a crouching action followed immediately by a jump for maximal height. Each subject completed five attempts. Two minutes of rest for complete recovery was given between jumps. The hands were on the hips throughout the entire jump. The best and the worst jumps were eliminated from the data, and the average of the other three jumps was recorded. The CMJ had an intraclass correlation coefficient (ICC) of 0.95 (0.92–0.97), and a coefficient of variation (CV) of 5.8%.

Subsequently, maximal power was assessed for loaded CMJ and full squat exercises. Power measurement for both exercises was accessed in a Smith Machine. The bar of the Smith Machine had a linear transducer attached (Globus Real Power, Italy). The rotary encoder of the linear transducer recorded the position and direction of the bar to within an accuracy of 0.0002 m. in order to calculate different variables for each repetition of the CMJ and full squat. Only the concentric phase of the movement was taken for further analysis. Concentric movement was defined from the moment following the end of the eccentric phase until maximal positive velocity was achieved. The CMJ weighted test began with a load of 20 kg (CMJ_20_), and the weight was increased with 10 kg increments. The test ended when CMJ height was less than 20 cm. This height was used as reference because a jump lower than 20 cm progressively decreased the reliability of the jump (Vitaasalo, 1985) and it increases the risk of injury. Four minutes of rest were provided between each attempt to minimize the likelihood of fatigue. The best attempt for peak power with each load was recorded for later analysis. The CV of variation for test-retest reliability for CMJ was 4.3% and less. In addition, the CMJ with additional weights showed an ICC of 0.93 or more.

The progressive test of full squats with external loads was performed on the Smith machine. For this test, each soccer player descended until the top of the thigh was below parallel with the floor. The number of full squat repetitions for a particular load was determined according to the velocity of the first repetition. Three repetitions were performed when the subject displaced the bar with an average velocity ≥ 1m·s^−1^. In contrast, only two repetitions were executed when the average velocity was <1m·s^−1^. The increase in the load was done in 10 kg increments. Four minutes of recovery were taken between each set. The test ended for each subject when the average velocity of movement was ≤ 0.7 m·s^−1^ (19). This velocity was chosen as reference because in a pilot study it was observed that maximum average power was attained at higher velocities. The highest peak power and mean power of the repetitions done with external loads and the load achieved by each player were used for later analysis (CV: 2.9–4%; ICC: 0.92–0.94).

Finally, subjects were required to perform three maximum effort sprints of 30 metres. Times at 0–10m (T_10_), 0–20m (T_20_), 0–30 (T_30_), 10–20m (T_10–20_), 10–30m (T_10–30_), and 20–30m (T_20–30_) were recorded using Brower equipment (Wireless Sprint System, USA). Subjects performed trial sprints separated by 3 minutes of rest. Only the best attempt was considered. The sprints reported ICCs of 0.92–0.99 and CVs of 1.2–2.6%.

### Statistical analyses

To assess the relationship between sprint performances and jump and full squat performances Pearson correlation and univariate analysis of variance between the different variables were used. Post hoc Bonferroni comparison was used to locate significant differences. Intraclass correlation coefficient (ICC) was used to determine between-subject reliability of jumping tests. Within-subject variation for all tests was determined by calculating the coefficient of variation (CV) as outlined by Hopkins (2000). Statistical significance was accepted at p ≤0.05 for all analysis.

## Results

Means values of the countermovement jump and 30m sprint performances are presented in Table 1.

**Figure 1:**
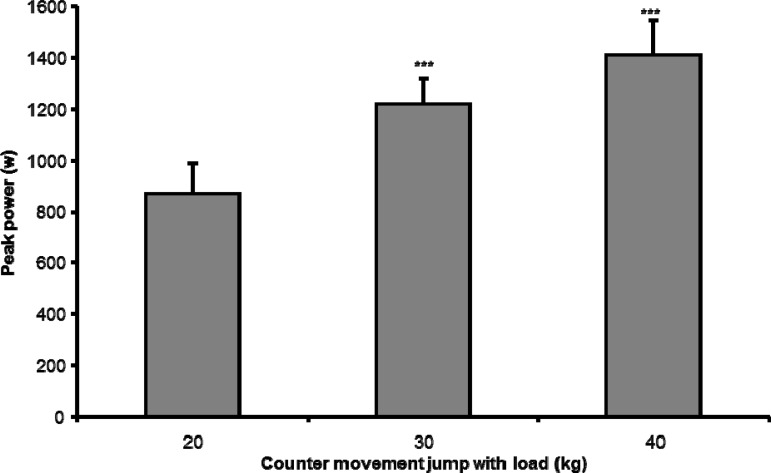
Average peak power and SD at the counter movement jumps with different weights (Significant difference in peak power output with previous load= *p ≤0.05; **p ≤0.01; ***p ≤0.001).

**Figure 2:**
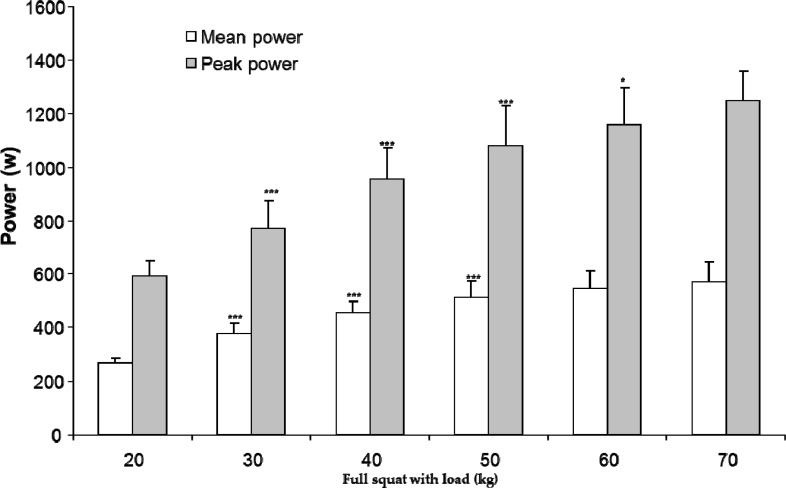
Average mean and peak power and SD at the full squats with different weights. (Significant difference in power output with previous load= *p ≤0.05; ***p ≤0.001).

Peak and mean power output showed significant differences with the increasing load in the countermovement jump and full squat (Figure 1 and 2) (i.e. the mean and peak power increased with increasing load).

The present results showed significant correlations between the countermovement jumping height and the sprint times at 20 and 30m. Peak power in CMJ test with an external load of 20kg correlated significantly with sprint times at every distance (Table 2). Finally, the last meters of the sprint (T10–30 and T20–30) were associated with peak power at different weighted CMJ. No correlations were found between the CMJ with 20 and 30kg load and the times at 10 and 20m.

Regarding the full squat, maximal average power was obtained with 62 ± 9kg, equivalent to 82% of players body mass. Furthermore, maximal peak power was attained with 65 ±12kg (86% of body mass). Only the peak power output with 20 and 30kg showed significant correlations with different sprint times (Table 3). The mean power in full squat with an external load of 70kg showed significant correlations with the majority of sprint times (Table 3). A significant correlation was also found between the time at 10m and the average power during the full squat with 30 and 40 kg external load. No other significant correlations were observed between sprint times and the power parameters during the full squat with different external loads (Table 3)

## Discussion

The purpose of this study was to investigate the relationships between short sprint performance and power parameters during vertical jump and full squat exercises in under-21 soccer players. The results showed significant correlations between all sprint times and peak power in loaded countermovement jump with 20kg, and between the loaded countermovement jumps and split times from 10 to 30m sprint. The average power with the full squat with 70kg also showed significant positive correlations with the sprint times.

The CMJ height has been greatly used to access lower body power in soccer players (Wisloff, 1998; Helgerud, 2001; Núñez, 2008; Ronnestad, 2008). Nevertheless, to our knowledge, only two previous studies (Gorostiaga, 2004; López-Segovia, 2010) have used loaded countermovement jump (CMJ_L_) exercise for testing lower limb power in this population. Unfortunately, these authors (Gorostiaga, 2004; López-Segovia, 2010) did not include sprint evaluations in their studies.

Different factors such as lower reliability of testing at very short distances, the static start position in the sprint test and the location of the first photoelectric cells (30 cm behind start in these two studies) could explain the lack relationship reported between CMJ and time at 10m. Although, the relationship obtained between the vertical jump and 30m sprint time (present study: r= −0.55; p<0.05 *vs.* r= −0.60; p<0.01) was similar to the study of Wisloff (2004), the relationships observed between the vertical jump and last running meters are consistent with the results perceived with loaded jump, given a similarity of muscle action in both types of jumps. Significant association between peak power during loaded CMJ and later stages of the sprint (r=−0.544 to −0.611; p≤0.05) were obtained. The T_10–30_ and T_20–30_ were significantly related with peak power observed in the CMJ_L_ exercise with 20, 30, and 40kg external load. Cronin and Hansen (2005) observed similar results in professional rugby players between loaded (30kg) vertical jump height and 5m, 10m, and 15m sprint times. The higher relationships (R^2^= 41–62%) observed in the present study were perceived with the longer distances rather than the initial run. As running velocity approaches maximum, those strength measures that require force to be produced at high velocities have been reported to be significantly related to sprint performance (Wilson, 1995; Young, 1995; Nesser, 1996). Wilson (1995) reported a significant relationship between force at 30 ms in a concentric squat jump and 30m sprint time (r= 0.62). Nesser (1996) claimed significant correlations between 40m sprint time and peak isokinetic torque at a velocity of 7.85 rad/s for the hip and knee extensors and knee flexors (r= 0.54 to 0.61). We agree with the assertion that results show a slight tendency of increased relationships such as velocity and distance increased (Table 2).

Moreover, data showed that power output during the vertical jump with 20kg best explained sprint performance. This parameter was also significantly correlated with all split speed measurements, including the first sprint stages. Although correlations do not signify causation, CMJ training with light loads could be important to improve sprint performance in soccer player’s under-21. McBride (2002) also observed that training with light loads (squat jump at 30% of 1RM vs. 80% of 1RM) was more effective to velocity improvement. Further, this type of strength training can reduced the risk of injuries compared to heavy resistance training regimens. Finally, this training method can be used with athletes of short resistance training experience as has beeen shown favorably in previous studies with soccer players (Gorostiaga 2004; López-Segovia 2010).

Only one previous study has investigated the muscular power output in the squat in soccer players (Requena, 2009). Yet, no studies have analysed the power output in the full squat exercise in this population. Maximal peak power output reported in the present study was 1181 ± 188W, with an external load equivalent to the 86% of body weight. This result was very similar compared to the values (1149W) claimed by Requena (2009) among semi-professional soccer players. However, these researchers used the traditional half squat exercise and not the full squat movement. Although similar peak power outputs (1181 vs. 1149) were reported between theses two studies, ours was equivalent to 86% of players body weight, while the experiment conduced with semi-professional soccer players was obtained with 112.5% of BW. These differences could be explained by a different range of motion of these two exercises (half vs. full squat), by different measuring equipment, and by distinct kinematic parameters. Thereby a load equivalent to 55% of 1RM in the half squat represents in a full squat approximately 30–45% of the subjects maximum capability (Baker 2001). It is also possible that the differences could be linked to the players experience in resistance-training programs. Baker (2001) observed that professional rugby players produced maximal peak power values with a load equivalent to 104% bodyweight, while in a group of college rugby players the percentage obtained was lower (92%), close to our results. Furthermore, both studies (ours and of Baker, 2001) were conduced with participants who had limited resistance-training experience. Nevertheless, these results seem to indicate that power output could be a determinant factor to identity full squat performance at different levels.

Like in loaded countermovement jump, the power output obtained in full squat with lighter loads was more related as running velocity approaches maximum (Table 3). These relationships are in accordance with the findings of Murphy (2003) showing that at initial acceleration, the application of reactive strength is longer and the stretch shortening cycle is slower that in the last meters of the sprint. The major relationships observed in the last sprint stages may also indicate individual ability to produce muscle force at higher velocities, and also a better neuromuscular activation during the stretch shortening cycle.

The current study showed that the T_10_ correlated significantly with the average power during the full squat with 30 and 40kg (r= −0.591, −0.621, and r=−0.602 respectively; p≤0.05). The relationships found between maximal strength variables (1RM or XRM) and initial run capacity is not well establish. In fact, only Wisloff (2004) reported significant correlations (r= 0.94–0.71, p≤0.01–0.001) between maximal strength and sprint times at 10m, and 30m. Other studies showed small or no significant correlations between lower body maximal strength and sprint performance (Baker and Nance, 1999; Cronin and Hansen, 2005; Harris, 2010). More recently, however, López-Segovia (2010) described significant correlations (r= 0.53–0.64, p≤0.05) between the changes in short sprint performance and the changes in the bar velocity and displacement in the full squat exercise after four months of resistance training combining soccer training and weekly competitions. It shows the importance of combined speed-strength velocity in full squat exercise as specific neural adaptations in order to improve sprint performance (McBride, 2002). In sport science, many experiments, set up to enhance the performance of the athlete, are based on a force-velocity relationship. The relationships between power and velocity and/or force are also a way of examining the force-velocity relationship from another point of view.

Interestingly, the present data also showed significant relationships between average maximal power during the full squat with 70kg and sprint times (r=−0.62 to −0.78; p≤0.05). This load was equivalent to 93% of body mass and very similar to the result reported by Requena (2009) with semi-professional soccer players. These researchers indicated that the load representative to 100% of body mass was significantly related to 15m sprint time (r=−0.62; p≤−0.01). These relationships might suggest that certain levels of neuromuscular activation, assessed by mean power output and produced versus loads very close to body weight and maximal power, are more related with sprint performance reflecting the greater suitability of loads against others for the improvement of this quality. Of great interest was that power output produced with this specific load (FS_70_) it’s near to maximal average power, and based in theoretical principles training with this range of loads may cause neural and muscle fiber adaptations related to sprint action (Wilson, 1993; Baker, 2001). Although the study’s limitations do not allow to verify the following possibility, the constant relationship found between FS_70_ and all split of sprint could be due to similar motor-unit recruitment and synchronization to type II muscle fibers, which are important neural factors to explosive movement production (Moritani, 1993).

In summary, power parameters obtained in a loaded jump with 20kg and full squat exercise with 70kg demonstrated significant relationships with short sprint performance in under-21 soccer players. Therefore, the results may suggest that power produced either with vertical jump or full squat exercises is an important parameter to explain short sprint performance in soccer players.

Although these relationships do not imply the causes, the neuromuscular adaptations provoked by light loads in exercises such as the squat and loaded vertical jump might have a significant effect on sprint ability, as previously documented (McBride, 2002). Additionally, using lighter weights diminishes the risk of injury.

### Practical applications

Power produced either with vertical jump or full squat exercises is an important parameter to explain short sprint performance in soccer players. This information could be useful for practitioners to monitor their strength training programs in order to improve sprint performance in under-21 soccer players. Future research with resistance training with light loads is required to elucidate the efficiency of this type of training focus on the importance of shortening capacity of the extensor muscles at higher velocities, against the traditional strength training focus on the high magnitude of load lifted.

## Figures and Tables

**Table 1:** Means ± SD of counter movement jump and 30m sprint performances in all tested subjects.

Variables	Values
Counter movement jump (cm)	38.34 ± 4.44
Time at 10 m (s)	1.92 ± 0.06
Time at 20 m (s)	3.22 ± 0.09
Time at 30 m (s)	4.43 ± 0.14
Split time between 10 and 20 m (s)	1.28 ± 0.04
Split time between 10 and 30 m (s)	2.50 ± 0.05
Split time between 20 and 30 m (s)	1.20 ± 0.05

**Table 2:** *Correlations between height in countermovement jump and peak power output in countermovement jumps with different weights, and the different sprint times*.

	CMJ	CMJ_20_	CMJ_30_	CMJ_40_
	Height		Peak power	
Time at 10 m	−.46	−.56*	−.27	−.44
Time at 20 m	−.54*	−.58*	−.37	−.46
Time at 30 m	−.55*	−.69*	−.58	−.61*
Split time 10–20 m	−.49	−.57*	−.62*	−.51
Split time 10–30 m	−.52	−.75†	−.79†	−.72*
Split time 20–30 m	−.48	−.75†	−.65*	−.72*

*CMJ = countermovement jump; CMJ_L_= counter movement jump with load. Significance:*

* p≤0.05;

† p≤0.01.

**Table 3:** Correlations between peak power and mean power of full squats with different weights and the different sprint times

	Full squat_20_		Full squat_30_		Full squat_40_		Full squat_50_		Full squat_60_		Full squat_70_	
	PP	MP	PP	MP	PP	MP	PP	MP	PP	MP	PP	MP
Time at 10 m	−.59	−.50	.47	−.62*	−.30	−.60*	−.21	−.44	−.31	−.45	−.21	−.63*
Time at 20 m	−.44	−.38	−.40	−.44	−.29	−.50	−.21	−.37	−.37	−.49	−.08	−.73†
Time at 30 m	−.58	−.48	−.5	−.50	−.36	−.55	−.28	−.43	−.37	−.51	−.03	−.78†
Split time 10–20 m	−.44	−.33	−.18	−.18	−.24	−.28	−.08	−.20	−.21	−.42	−.05	−.62*
Split time 10–30 m	−.63*	−.50	−.48	−.42	−.33	−.48	−.28	−.39	−.29	−.50	−.14	−.76*
Split time 20–30 m	−.64*	−.56*	−.58*	−.53	−.34	−.55	−.35	−.45	−.30	−.49	−.07	−.71*

*Full squat_L_= full squat with load; PP= peak power, MP=mean power Significance:*

* p≤0.05;

† p≤0.01
